# Impact of conditional economic incentives and motivational interviewing on health outcomes of adolescents living with HIV in Anambra State, Nigeria: A cluster-randomised trial

**DOI:** 10.1016/j.conctc.2022.100997

**Published:** 2022-09-15

**Authors:** Obinna Ikechukwu Ekwunife, Maureen Ugonwa Anetoh, Stephen Okorafor Kalu, Prince Udegbunam Ele, Bolaji Emmanuel Egbewale, George Uchenna Eleje

**Affiliations:** a Department of Clinical Pharmacy and Pharmacy Management, Nnamdi Azikiwe University, Awka, Nigeria; b Virology Laboratory, Nnamdi Azikiwe University Teaching Hospital, Nnewi, Nigeria; c Division of Respiratory Medicine, Department of Medicine, Nnamdi Azikiwe University Teaching Hospital, Nnewi, Nigeria; d Department of Community Medicine, College of Health Sciences, Ladoke Akintola University of Technology, Ogbomoso, Nigeria; e Department of Obstetrics and Gynaecology, Nnamdi Azikiwe University Teaching Hospital, Nnewi, Nigeria

**Keywords:** Adolescence, HIV, AIDS, Conditional cash transfer, Financial incentives, Motivational interviewing, Nigeria, Cluster-randomised trial

## Abstract

**Background:**

Adolescents living with HIV (ALHIV) have had worse outcomes compared to adults. They face enormous difficulty in accessing HIV care services. We hypothesize that conditional economic incentives (CEI) and motivational interviewing could increase retention in care, medication adherence and ultimately viral load suppression. Therefore, we evaluated the one-year impact of conditional economic incentives and motivational interviewing on the health outcomes of ALHIV in Anambra State, Nigeria.

**Methods:**

Using a cluster-randomised design, we examined the one-year (from December 1, 2018, to November 30, 2019), individual-level impact of an Incentive Scheme comprising conditional economic incentives and motivational interviewing on achieving undetectable viral load (primary outcome), CD4^+^ count, adherence to antiretroviral therapy and retention in care (secondary outcomes) by ALHIV in Anambra State, Nigeria. Twelve HIV treatment hospitals were stratified according to the type of clinic (secondary or tertiary) and randomly assigned to the intervention arm or control arm to receive the Incentive Scheme or routine care, respectively. ALHIV aged 10–19 years, initiated into HIV care for a minimum of 6 months, and who adhered poorly to medications (<100% adherence rate) were eligible for the study. Participants in the intervention arm received motivational interviewing at the study baseline and every visit. They also received US$5.6 when HIV viral load (VL) was <20 copies/mL at month 3, US$2.8 if the VL remained suppressed at months 6 and 9, and US$5.6 if the VL remained <20 copies/mL at month 12.

**Results:**

Of the 246 trial participants, 119 were in the intervention while 127 were in the control arm. There was no difference in the baseline characteristics of the participants between the intervention and control arm except for the number of participants with undetectable viral load and the number of participants with ≥95% adherence. Although participants in the intervention arm had a 10.1% increase while those in the control arm had a 1.6% decrease in proportion with undetectable viral load (≤20 copies/ml) after 12 months, the change in the primary outcome was not statistically significant. Similarly, the differences in the secondary outcomes were not statistically significant.

**Conclusion:**

The Incentive Scheme did not improve the virologic outcome of ALHIV after 12 months. Differences in the secondary outcomes after 12 months were also not significantly different from the baseline.

**Trial registration:**

We registered the trial retrospectively with The Pan African Clinical Trials Registry: https://pactr.samrc.ac.za/(PACTR201806003040425) on 2/2/2018.

## 1 Background

Adolescents living with HIV (ALHIV) are associated with poor treatment outcomes [1,2]. A longitudinal study in Nigeria found that the proportions of ALHIV lost to follow-up were 19.1% during adolescence and 13.7% during the transition to adult-centred care and that their viral load suppression rates through adolescence and post-transition were only 55.6%–64.0% [3]. Tremendous physical, cognitive, emotional, growth, and social challenges, significantly influencing ALHIV psychological and health needs, could cause their poor retention in care and adherence to ART, consequently leading to poor virologic outcomes [4]. Adolescence is a unique stage of human development and is usually associated with a desire for self-discovery, independence, recognition, and acceptance. Additionally, structural barriers such as school attendance may affect their retention in care. Therefore, practical and adolescent-friendly interventions to support the retention of ALHIV in hospital care and ensure their adherence to ART are essential to improve the health outcomes of ALHIV and reduce transmission of HIV. Such service delivery intervention is necessary to achieve the United Nation's Sustainable Development Goals and 95-95-95 targets for 2030 [5].

A systematic review has shown that offering individual and group education and counselling, financial incentives, increasing clinic accessibility, and provision of specific adolescent tailored services appeared to be promising interventions to support adolescents’ linkage from HIV diagnosis to ART initiation, retention in care and ART adherence [6]. Task shifting, community-based adherence support, mobile health (mHealth) platforms, and group adherence counselling have been identified as promising interventions to support retention in care and adherence to antiretroviral therapy among ALHIV [7]. Additionally, decentralization, down-referral of stable patients, task-shifting of services, differentiated treatment, and retention in care among adults were found to have statistically significant relationships in another systematic review [8].

We, therefore, examined the impact of two selected interventions identified by the systematic reviews on treatment outcomes among ALHIV in the Nigerian context. Our choice of conditional economic incentive (CEI) and motivational interviewing has some theoretical basis. Adherence to antiretroviral therapy relates to a perception of reduced utility since there is out-of-pocket cost associated with hospital visits, side effects related to antiretroviral treatment, and delayed benefits from antiretroviral therapy [6]. CEI helps increase utility related to antiretroviral treatment since meeting set conditions increases income. CEI provides an immediate and observable benefit to consistent medication-taking and may help improve adherence initially [9]. However, the impact of CEI may not be sustained, especially after the withdrawal of the financial incentives. For instance, the Nigeria Subsidy Reinvestment and Empowerment Programme (SURE-P) Maternal and Child Health (MCH) program used CEI to increase demand and access to maternal and neonatal health services, but needed to track the beneficiaries to sustain its impact [10]. Therefore, we added motivational interviewing to engender habitual behaviour change. Motivational interviewing (MI) is a counselling technique based on self-determination theory and is recognized as a pragmatic way of getting people to change their behaviour by augmenting their internally motivated change process [11]. In a quasi-experimentally designed study, CEI combined with motivational interviewing impacted positively the health outcomes of ALHIV [12].

Therefore, this trial used a cluster randomised controlled trial (RCT) to evaluate the one-year impact of conditional economic incentives and motivational interviewing on achieving undetectable viral load, retention in care, and adherence to antiretroviral drug therapy by ALHIV in Anambra State, Nigeria.

## 2 Methods

### 2.1 Trial design

The trial was a cluster randomised controlled trial and was conducted between 1st December 2018 and 30th Nov 2019. The use of cluster randomisation was practical and avoided treatment group contamination. Clusters were HIV treatment hospitals in Anambra State, located in south-eastern Nigeria. According to the National Bureau of Statistics, the primary indigenous ethnic group in Anambra State is Ibo. There are 21 local government areas in the state with an estimated population of 4.1 million. Anambra State has a 95.7% youth literacy school. As of 2019, Anambra State had an HIV prevalence of 2.7% (4th highest in the country) [13]. We matched the hospitals by type of hospital (e.g. secondary or tertiary), and randomly assigned the paired units to the intervention or the control arm. The trial had two periods – the intervention period and the post-intervention period. The result of the pre-intervention period is presented in this report. The trial was registered with The Pan African Clinical Trials Registry: https://pactr.samrc.ac.za/(PACTR201806003040425) on 2/2/2018 and implemented according to a published protocol [14]. The following sections summarise the trial protocol.

### 2.2 Trial sites selection

We purposively selected twelve hospitals with appreciable client load that offer complete HIV services and registered with the National Agency for the Control of AIDS (NACA) in Anambra State. Seven hospitals were private (3 in intervention and 4 in control arm), while the other five were government-owned (3 in intervention and 2 in control arm). The matched hospitals (the clusters) were randomly assigned to intervention or control [14].

### 2.3 Participants

Eligibility criteria for participants were: all adolescents with HIV; 10–19 years irrespective of CD4^+^ cell count; initiated into HIV care and antiretroviral therapy for a minimum of 6 months; and sub-optimal medication adherence (<100% adherence rate or missed one tablet in the last month assessed through pill count or self-report if the former was not feasible, i.e. participant forgot to come with his pill container) [14].

### 2.4 Intervention

A structured adherence support scheme termed the 'Incentive Scheme' was applied to the intervention hospitals while the control hospitals received routine care. The Incentive Scheme added to the standard care were conditional economic incentives linked to participant achieving undetectable viral load, checked every quarter in the intervention arm, combined with attendance to motivational interviewing administered individually during a monthly scheduled hospital visit with staff other than the adherence counsellors trained in motivational interviewing techniques [15]. We selected the CEI amount based on the daily minimum wage since the monthly minimum wage in Nigeria is NGN 30,000 (US$83) or NGN 1000/day (US$ 2.8/day) [16]. A hospital staff (nurse or pharmacist) was trained in each of the hospitals in the intervention arm to deliver motivational interviewing (MI) following a guide that covered key points and contained examples and critical insights to assessing participant risk, risk reduction counselling and inspiring behaviour change [17]. We conducted individualised training for each hospital in the intervention arm and recapitulated during the site initiation visit before trial commencement.

Participants in the intervention hospitals (alone or with a guardian) received MI at baseline and every hospital visit. Each participant was expected to attend six MI sessions and each session lasted for about 15 min. Additionally, they received direct Cash of US$5.6 when VL was <20 copies/mL at month 3, Cash of US$2.8 if the VL remained suppressed at months 6 and 9, and finally Cash of US$5.6 if the VL remained <20 copies/mL at month 12 (Table 1). All cash incentives were conditional on participants meeting their VL target and attending motivational interviewing. Those with VL higher than the thresholds did not receive the incentive. The potential maximum cumulative financial incentive was US$16.8, with a sustained undetectable viral load for 12 months. The participants had the choice to withdraw from the scheme at any time. Participants in hospitals randomised to usual care received the routine maintenance obtainable in the HIV treatment hospitals, which included scheduled hospital visits every three months for stable participants and monthly or bi-monthly hospital visits for non-stable participants for medical examination, prescription refill and adherence counselling, yearly VL and 6-monthly CD4^+^ count assessments where viral load is not available [18].

**Table 1:** Protocol for conditional economic incentives.

	VL response and attended motivational interviewing	Economic incentive (US$)
Month 3	VL ≤ 20 cells/mL	5.6
Month 6	Sustained VL ≤ 20 cells/mL	2.8
Month 9	Sustained VL ≤ 20 cells/mL	2.8
Month 12	Sustained VL ≤ 20 cells/mL	5.6
Total		16.8

VL – Viral load.

Exchange rate - US$1 = 360 Nigerian Naira.

We collected routine hospital data and assessed outcomes by changes in HIV VL and CD4^+^ count after 12 months. Each study subject was assigned to a study nurse/pharmacist working in the HIV treatment hospital who tracked them with their mobile phone numbers based on hospital protocol.

### 2.5 Outcomes

The trial's primary outcome was the difference between groups in proportion with undetectable VL (≤20 copies/mL) by 12 months. The secondary outcome measures were the average change in CD4^+^ count, the difference between groups in proportion with ≥95% adherence (measured using pill count only), and retention in care defined as persons with diagnosed HIV who had at least two medical visit dates that were at least 90 days apart in the measurement year [19]. We measured the primary and secondary outcomes at the level of an individual adolescent.

### 2.6 Sample size

We used a fixed number of six clusters or hospitals per arm (i.e. a total of 12 hospitals in the trial) because only 12 hospitals in Anambra State offered comprehensive HIV services, including HIV-adherence counselling and antiretroviral treatment services at the time of the study initiation and these 12 hospitals had appreciable HIV client load. We estimated the sample size using a web-based calculator of the University of Califonia San Francisco (UCSF) Clinical and Translational Science Institute [20]. We based the sample size calculation on the proportion of participants with undetectable VL ≤ 20 copies/mL (the primary outcome measure). Based on a power of 80% and an α of 0.05 (two-sided), 63 participants per group were needed to observe a 12% (assumed standard deviation of 24%) increase in the number of participants with undetectable VL as previously reported [14]. After adjustment for the cluster design, based on the assumed intracluster correlation coefficient of 0.047 and a fixed cluster per arm of six HIV treatment hospitals, the adequate sample size increased to 120 participants per arm (i.e. a total of 240 participants). Due to potential attrition that could arise as a result of severe adverse events, treatment failures or the participant simply deciding to withdraw, we added three participants per hospital, i.e. 15% of the calculated sample size, to increase the number of participants in each arm to 138 (i.e. a total of 276 participants). Each of the hospitals had a target to recruit 23 participants.

### 2.7 Randomisation

We stratified each cluster unit (HIV treatment hospital) according to the type of clinic (secondary or tertiary). We randomly allocated each HIV treatment hospital into the intervention or control arm in each stratum. Research Randomizer, a web-based computer random-number generator, was used to generate the randomisation schedule [21]. An independent person who was not part of the research team carried out the randomisation schedule and assigned clusters to either intervention or control. The principal investigator kept the treatment allocation for each trial site until the completion of the phlebotomists' general training. The site doctor and nurse or pharmacists were trained on-site after treatment allocation.

### 2.8 Statistical analysis

We used descriptive statistics to present the differences in demographic and clinical characteristics with descriptive statistics. The analysis was based on intention-to-treat. We used a single imputation method (last observation carried forward) in line with a prespecified analysis plan to treat missing data. All observations were analysed in the arm they were randomised. We analysed all outcomes using the Poisson multilevel regression model with HIV treatment hospital as a random effect for estimation of the incidence risk ratio for dichotomous variable and mean difference for continuous variable. We adjusted baseline measures of the primary outcome variable, age, and sex of study participants in the model. In the analysis, usual care was the reference category. A *p-*value of <0.05 was used to indicate statistical significance. The analysis was conducted with Stata (version 17).

## 3 Results

### 3.1 Trial participants' baseline characteristic

**Fig. 1:**
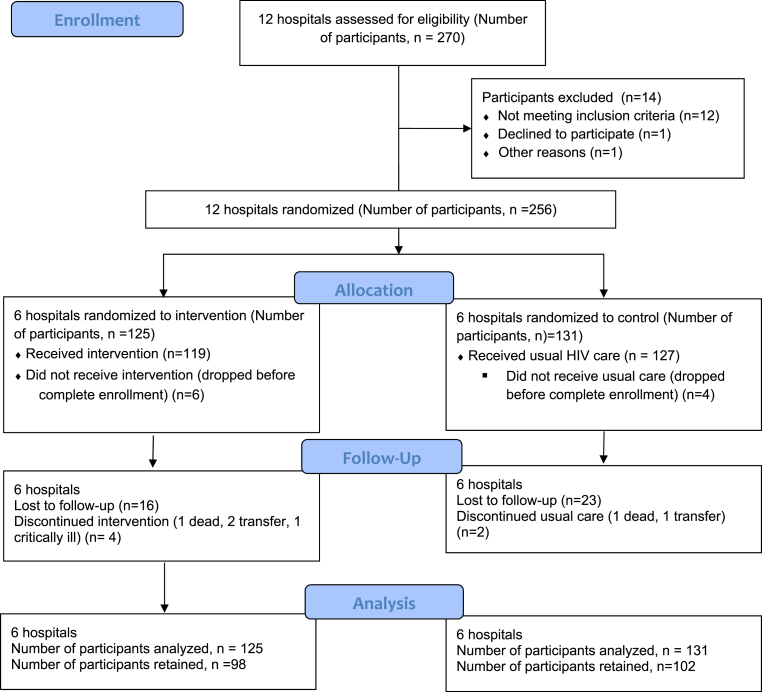
Trial flow diagram.

The intervention and control arm participants were 119 and 127, respectively (Fig. 1). Participants were followed up for one year (i.e. until February 28, 2020, for the last recruited participant). The mean age and the gender of participants in both arms were not statistically different (13.7 ± 2.4 versus 14.0 ± 2.7, p = 0.35 for intervention versus control respectively). More participants in the intervention arm received more Zidovudine/Lamivudine/Nevirapine regimen than those in the control arm. Conversely, more participants in the control arm received Tenofovir/Lamivudine/Efavirenz and Tenofovir/Lamivudine/Dolutegravir combinations compared to those in the intervention arm. More participants in the control arm had an undetectable viral load (≤20 copies/ml) compared to those in the intervention arm at baseline (42.5% versus 2.18%, p < 0.001 respectively). There was no statistically significant difference in the CD4^+^ count of the participants in the two arms at baseline (665 ± 685 versus 665 ± 437, p = 0.99 for intervention versus control respectively). More participants in the intervention arm had ≥95% adherence to antiretroviral therapy compared to those in the control arm (51.3% versus 27.6%, p < 0.01 for intervention versus control respectively). The details of the baseline characteristics of participants are shown in Table 2.

**Table 2:** Baseline characteristics of trial participants (N = 246).

	Intervention	Control	*P*-value
Number of participants	119 (48.4%)	127 (51.6%)	0.61
Mean age of participants ± Std. deviation	13.67 ± 2.43	13.98 ± 2.72	0.35
Gender of participants			
Male	63 (52.9%)	60 (47.2%)	0.44
Female	56 (47.1%)	67 (52.8%)	
ART Regimen at baseline			
*Zidovudine/Lamivudine/Nevirapine*	67 (56.3%)	45 (35.4%)	–
*Tenofovir/Lamivudine/Efavirenz*	32 (26.9%)	43 (33.9%)	
*Tenofovir/Lamivudine/Dolutegravir*	5 (4.2%)	16 (12.6%)	
*Abacavir/Lamivudine/Lopinavir/ritonavir*	2 (1.7%)	8 (6.3%)	
*Zidovudine/Lamivudine/Lopinavir/ritonavir*	2 (1.7%)	7 (5.5%)	
*Tenofovir/Lamivudine Lopinavir/ritonavir*	5 (4.2%)	4 (3.1%)	
*Abacavir/Lamivudine/Efavirenz*	5 (4.2%)	3 (2.4%)	
*Zidovudine/Lamivudine/Efavirenz*	1 (0.8%)	1 (0.8%)	
Number of participants with undetectable viral load (≤20 copies/ml)	26/119 (21.8%)	54/127 (42.5%)	<0.001
Mean CD4^+^ count ± Std. deviation	665 ± 685	665 ± 437	0.99
Number of participants with ≥95% adherence	61/119 (51.3%)	35/127 (27.6%)	<0.001

Abbreviations.

Zidovudine/Lamivudine/Lopinavir/ritonavir.

Tenofovir/Lamivudine Lopinavir/ritonavir ART Antiretroviral therapy.

### 3.2 Impact of incentive scheme after 12 months

The unadjusted impact of the incentive scheme on health outcomes after 12 months is shown in Table 3. There was a 10.1% point increase in the number of participants with undetectable viral load (≤20 copies/ml) in the intervention arm, while a 1.6% point decrease was observed in the control arm. The mean CD4^+^ count decreased more in the intervention arm than in the control arm. Also, the control arm had a greater number of participants achieving ≥95% adherence than the intervention arm. Lastly, participants in the intervention arm achieved higher retention in care than those in the control arm (Table 3).

**Table 3:** Unadjusted primary and secondary outcomes at 12 months (N = 246).

	Intervention			Control		
	*Baseline*	*At* 12 months	*Change over* 12 months	*Baseline*	*At* 12 months	*Change over* 12 months
Number of participants with undetectable viral load (≤20 copies/ml)	26/119 (21.8%)	38/119 (31.9%)	10.1%	54/127 (42.5%)	52/127 (40.9%)	−1.6%
Mean CD4^+^ count ± Std. deviation	665 ± 685	587 ± 379	−78	665 ± 437	611 ± 387	−54
Number of participants with ≥95% adherence	61/119 (51.3%)	59/119 (49.6%)	−1.7%	35/127 (27.6%)	40/127 (31.5%)	3.9%
Number of participants retained in care	–	98/119 (82.4%)	–		102/127 (80.3%)	–

### 3.3 Adjustment of study outcomes for baseline differences

Table 4 shows the incidence risk ratio (IRR) or mean difference for the primary and secondary outcomes at 12 months adjusted for baseline viral load, gender and age of participants in the two arms. There was no significant difference after 12 months on the number of participants with undetectable viral load (Incidence risk ratio, IRR = 1.01, p-value = 0.96), number of participants with ≥95% adherence (IRR = 0.69, p = 0.10), number of participants retained in care (IRR = 1.03, p = 0.79) and mean CD4^+^ count (IRR = 0.79, p = 0.92) between the intervention and treatment arm.

**Table 4:** Adjusted primary and secondary outcomes at 12 months using Poisson multilevel regression analysis.

Variables	IRR	P-value	95% Conf. Interval
Number of participants with undetectable viral load (≤20 copies/ml)	1.01	0.96	0.72–1.41
Number of participants with ≥95% adherence	0.69	0.10	0.45–1.07
Number of participants retained in care	1.03	0.79	0.78–1.38
	**MD**	**P-value**	**95% Conf. Interval**
Mean CD4^+^ count	0.79	0.92	−14.53 – 16.12

IRR – Incidence risk ratio. MD – Mean difference. Usual care was the reference category. The final multilevel analysis was adjusted for baseline viral load, gender (females versus males), and age (10–14 yrs versus 15–19 yrs).

### 3.4 Management of incidental findings

All anticipatable incidental findings due to the increased number of laboratory testing, which included clinical failure, immunological failure, and virologic failure, were disclosed to the participants and their parents/legal guardians (for those less than 18 years) and managed following the National Guidelines for HIV Prevention, Treatment, and Care [18].

## 4 Discussion

This cluster randomised controlled trial evaluated the impact of conditional economic incentives, linked with attending motivational interviewing on the primary outcomes (undetectable viral load) and secondary outcomes (CD4^+^ count, ≥95% adherence, retention in care) of ALHIV during a one-year intervention in Anambra State, Nigeria. The result showed that the change in the primary outcome was not statistically significant. Differences in the secondary outcomes after 12 months were also not significantly different.

Some studies examining the effect of conditional cash transfers or economic incentives on HIV outcomes arrived at different conclusions from our present study. Farber et al. showed that a monetary payment of USD 100 to adult participants dependent on either an undetectable viral load or having a viral load at least log10 lower than the lowest initial test increased the proportion of undetectable viral load test from 57% before the intervention to 69% at 12-month follow up (*p* = 0.03) [12]. In another study, adult participants in the intervention arm received about 15 vouchers for groceries or household items (USD 4–8 in values) earned for prespecified actions like initiating antiretroviral therapy, clinical/medication refill visits, viral suppression, completed monthly clinical follow-up visits compared to those in the control arm after 12 months [22]. A study in Congo that gave a cash payment conditional on adult women attending scheduled clinic visits and completing associated actions like providing a blood sample resulted in intervention participants being more likely to be retained in care six weeks post-partum compared to control participants [23]. Other studies have shown that conditional economic incentives improved the health outcomes of study participants living with HIV [[24], [25], [26]].

The difference in conclusion between these studies compared to our study could be due to the study setting (Anambra State) as structural interventions may not necessarily work in the same way in all settings. School attendance, for instance, is a factor that may affect adolescents’ ability to access care despite conditional cash incentives. Most adolescents in our study attend school during clinic hours, and some live in boarding schools. Most require travelling a long distance (with their parents in some cases) to access care in the comprehensive HIV treatment centres. Some of the parents being indigent needed to work to earn their living and were not disposed to accompany the adolescents to the hospital in some cases. These factors made it difficult for some adolescents to access care even with conditional economic incentives. That is why an adolescent health clinic that operates at different times from the general clinic has been advocated to provide for their specific developmental needs, including schooling and privacy [27]. Also, the hospital where the intervention is implemented determines the effectiveness of that intervention. Although not part of our study objective, we observed that private hospitals (predominantly in the control arm) performed better than government-owned hospitals (predominantly in the intervention arm) in implementing the trial protocol and in retaining participants. A Nigerian-based study found that private hospitals in Nigeria performed better than public hospitals, particularly in dignity, waiting times, and travel times [28].

While we hypothesised that conditional economic incentives could improve HIV health outcomes in the short term, the effect may not be sustained, especially as the incentives cease. Therefore, we added motivational interviewing to bring about a long-lasting effect. Motivational interviewing brings about internalised behaviour which helps in improving adherence to antiretroviral therapy and retention in care in the long term. Long-term adherence to antiretroviral treatment in adults has been linked strongly to internal motivation [29]. Also, the uniqueness of combining conditional economic incentives and motivational interviewing is due to limited funding. Given the decline in HIV funding (US$ 7.2 billion short of the US$ 26.2 billion UNAIDS estimates needed in 2020), it is essential to implement only efficient service delivery interventions in HIV care [30]. Since conditional economic incentive is applied for a short period, fewer financial resources are consumed. Foster et al. have shown the potential of combining financial incentives and motivational interviewing, although the UK study was a pilot study conducted on only eleven young adolescents [24].

There are some limitations in our trial that should be considered while interpreting our results. Some hospitals had a low client load and could not recruit up to the expected sample size (23 ALHIV). Thus, the trial did not reach the desired sample size. Given that retention in care was one of the trial outcomes, we could not influence retention in the study beyond what is done routinely in the hospitals used for the trial. The number of participants with undetectable viral load in the intervention arm was significantly lower than in the control arm at baseline. Also, the proportion of study participants on non-nucleoside reverse transcriptase inhibitor (NNRTI) was higher in the intervention arm than in the control arm. During the trial, there was a policy to change the drug regimen from efavirenz-based first-line therapy to dolutegravir-based first-line therapy [31]. Dolutegravir is equivalent to or superior to existing treatment regimens in both treatment-naïve and treatment-experienced subjects [32]. Its consistent efficacy, excellent tolerability and rare drug-drug interaction make the co-formulation of dolutegravir with two nucleotide reverse-transcriptase an appealing treatment choice [32]. The regimen transition affected participants in the intervention arm slightly more than those in the control arm. These three factors could have affected the impact of the intervention. Additionally, using pill count as an adherence measure has some limitations. Pill count is known to overestimate adherence and, at the same time, has the potential to underestimate adherence when refills are obtained earlier before their previous supply was depleted [33]. Participants could also remove the remaining pills from their pillboxes before the hospital visit.

Despite these limitations, the cluster randomised controlled trial design attempted to measure the impact of the incentive scheme on HIV health outcomes of ALHIV. Our findings highlight the need for implementation studies to evaluate an intervention's effect in a particular setting before its recommendation. For future studies, other implementation outcomes (e.g., acceptability and fidelity) as well as service outcomes (e.g. cost-effectiveness) and client outcomes (e.g. satisfaction) should be measured to fully understand the implementation process and compare the relative efficacy of the intervention with alternative strategies [34]. Also, factorial design is suggested in future studies to tease apart the impact of the two interventions combined in the Incentive Scheme. This will make it clear the intervention that added value.

## 5 Conclusion

Our study showed that conditional economic incentives and motivational interviewing did not improve the health outcomes of ALHIV in Anambra State after 12 months. These findings highlight the need for implementation studies to fully understand the implementation process of an intervention and enable the comparison of its efficacy with alternative competing strategies before adoption.

### Ethics approval and consent to participate

The study was conducted according to the Helsinki declarations on ethical principles from medical research involving human participants [35]. The study protocol was approved by Nnamdi Azikiwe University Teaching Hospital Ethics Committee NAUTH/CS/66/VOL.11/092/2018/052. For children below 12 years, informed consent of either parent or the parent that has primary responsibility for the child at the time of research or the legal guardian was obtained before enrollment. For children between 12 and less than 18 years, informed consent was obtained from the parent as described above while the child gave assent. Informed consent was obtained for those 18 years and older. In cases where the parents or legal guardians were not physically present, verbal informed consent through telephone calls was obtained. All informed consent was obtained after randomisation. Unique identifiers and a password-protected database were used to protect the personal information of the study participants. Participants' data were domiciled with the principal investigator. Participants were free to purposely leave the study at any time, without any effect on the care received in the study hospital.

## Authors' information

OIE is a Reader in the Department of Clinical Pharmacy and Pharmacy Management, Nnamdi Azikiwe University, Awka, Nigeria. He is also the coordinator of the Research Group for Evidence-Based Public Health (EBHC-UNIZIK), Nnamdi Azikiwe University, Nigeria. MUA is a PhD candidate and an academic staff of the Department of Clinical Pharmacy and Pharmacy Management, Nnamdi Azikiwe University, Awka, Nigeria. She is also a member of EBHC-UNIZIK. SK is the head of the virology laboratory, at Nnamdi Azikiwe University Teaching Hospital, Nnewi, Nigeria. PUE is the Head of the Respiratory Division, Department of Medicine and former Project Co-ordinator HIV CARE Department, Nnamdi Azikiwe University Teaching Hospital, Nnewi, Nigeria. BEE is an Associate Professor of Biostatistics in the Department of Community Medicine, Ladoke Akintola University of Technology, Ogbomoso, Nigeria. GUE is a Reader at Nnamdi Azikiwe University, Awka, Nigeria and an honorary consultant in the Department of Obstetrics and Gynaecology, Nnamdi Azikiwe University Teaching Hospital, Nigeria.

## Authors' contribution

OIE, SK and GUE designed the trial. MUA and PUE contributed to specific aspects of the trial design. OIE, MUA and PUE were directly involved in the implementation of the trial. SK and GUE had oversight functions for the trial. BEE conducted the statistical analysis of the study. OIE and MUA drafted the first manuscript. All authors participated in reviewing the manuscript. All authors read and approved the final version of the manuscript.

## Funding

This study is part of the EDCTP_2_ programme supported by the 10.13039/501100000780European Union (grant number TMA2016CDF-1548). The views expressed in this publication are those of the author(s). The funders had no role in the design of the study, analysis and interpretation of the data or writing of the manuscript.

## Consent for publication

Not applicable.

## Declaration of competing interest

The authors declare that they have no competing interests.

## Data Availability

The datasets generated and/or analysed during the current study are available in the Mendeley Data repository, https://data.mendeley.com/datasets/hjxs9s6g6n/1.

## List of abbreviations

AIDS: Acquired Immunodeficiency Syndrome
ART: Antiretroviral therapy
CEI: Conditional economic incentive
HIV: Human immunodeficiency virus
HRSA: Health Resources and Services Administration
LMIC: Low and middle-income countries
MCH: Maternal and Child Health
NACA: The National Agency for the Control of AIDS
NNRTI: Non-nucleoside reverse transcriptase inhibitor
PACTR: The Pan African Clinical Trials Registry
RCT: Randomised controlled trial
SURE-P: Nigeria Subsidy Reinvestment & Empowerment Programme
UNAIDS: The Joint United Nations Programme on HIV/AIDS
VL: Viral load
